# Supplementary material for: In Silico Investigation of the Human GTP Cyclohydrolase 1 Enzyme Reveals the Potential of Drug Repurposing Approaches towards the Discovery of Effective BH4 Therapeutics
Source: Int J Mol Sci. 2023 Jan 7;24(2):1210. doi: 10.3390/ijms24021210 (PMC9862521; doi:10.3390/ijms24021210)
Supplement: Supplementary file 1 [file ijms-24-01210-s001.zip › ijms-2070896-supplementary.pdf]

Structure	PBD code	Method of structure determination	Resolution Angstrom	Ligands
GCH1 Bacterial	1N3S	X-ray crystallization	2.55	GTP
GCH1 Human	1FB1	X-ray crystallization	3.01	Zinc
GCH1 Human	6Z86	X-ray crystallization	2.21	Zinc, 7-deaza GTP
GCH1 Human	6Z88	X-ray crystallization	2.69	Zinc, 5-azanyl-[1,3]thiazolo[5,4-d]pyrimidine-2,7-dione
GCH1-GFRP stimulatory complex Human	6Z80	EM	3	Zinc, Phenylalanine, 8-oxo-GTP
GCH1-GFRP inhibitory complex Human	6Z85	EM	2.9	Zinc, 7,8-dihydrobiopterin
GFRP Human	7ACC	X-ray crystallization	2.04	Potassium

Table S1. List of protein structures used for docking studies. EM: electron microscopy, GTP: guanosine triphosphate.

Library	Number of Compounds	Database	Description
FDA approved	1,500	Zinc	FDA approved drugs
Other (not FDA) Approved	4000	Zinc	Worldwide approved drugs non-FDA
Natural products	1,500	Zinc	Approved natural products with *in vivo* efficacy
FDA approved	1100	DrugBank	FDA approved drugs
Worldwide (not FDA) approved	3,440	DrugBank	Worldwide approved drugs non-FDA
Naturaceutical	74	DrugBank	Natraceutics
Comprehensive library (composed of all compounds listed above*)	7074	DrugBank and Zinc	All FDA and worldwide approved medications and natural products in addition to natural products with verified physiological activity

Table S2. Virtual screening library of compounds *repeated structures excluded

Drug Name	Category	Interactions with key residues	Docking Score kcal/mol	Docking Pose
7-deazaGTP	Inactive substrate	H bonds, salt bridge, Pi stacking, hydrophobic bonds observed with the following key binding residues: ARG 97, ASN 118, HIE 144, SER 166, LEU 163, LYS 167, ARG 170, GLN 182, GLU 183, HIE 210, ARG 216	-14.94	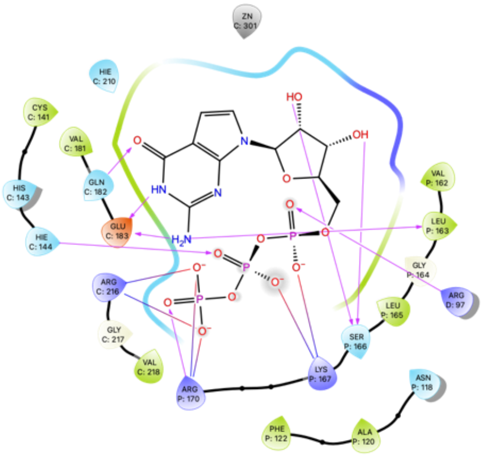
Polydatin	Natural product	H bonds, Pi stacking, hydrophobic GLN 182, GLU 183, HIE 210	-10.51	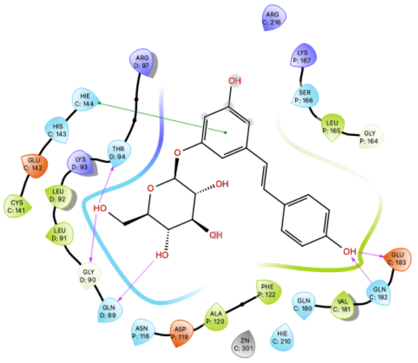
Fdump	Cytotoxic anti-neoplastic	H bonds, Pi stacking, hydrophobic bonds and salt bridges HIE 144, SER 166, LYS 167, ARG 170, ARG 216	-10.29	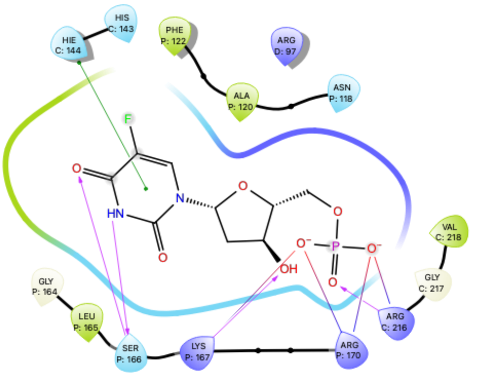

thioxanthyllic acid	Metabolite of azathioprine immunosuppressant	H bonds, Pi stacking, and salt bridges HIE 144, SER 166, LYS 167, ARG 170, ARG 216	-10.01	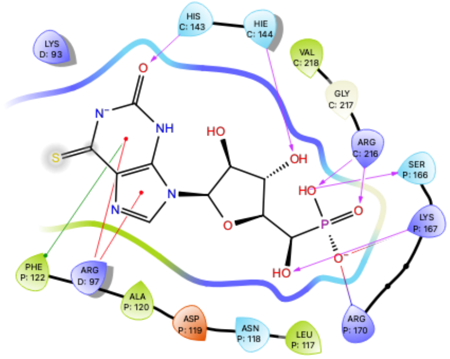
Phlorofucofuroeckol	Natural product Anti-cancer activity	H bonds, Pi cation, hydrophobic bonds ARG 97, ASN 118, SER 166, LYS 167, GLN 182, GLU 183, ARG 216	-10.02	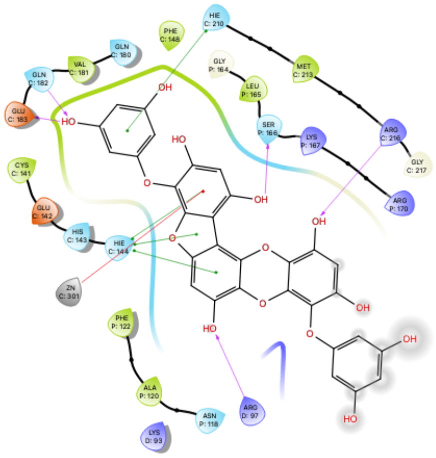
Fosphenytoin	Antiepileptic	H bonds, Pi stacking, Pi cation, hydrophobic ARG 97, HIE 144, LYS 167, ARG 170, ARG 216	-9.96	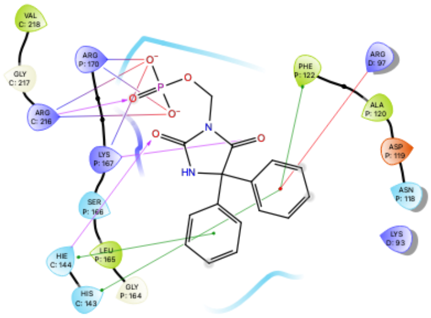
Masprocol	antineoplastic	H bonds, hydrophobic bonds ARG 97, ASN 118, LYS 167, GLN 182, GLU 183	-9.83	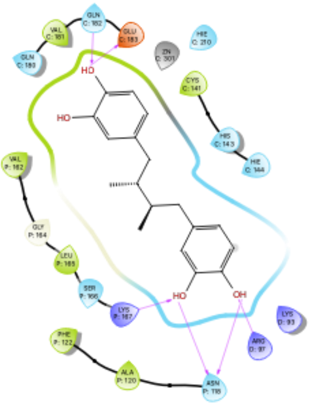

Penciclovir	Antiviral	H bonds, salt bridge, hydrophobic ASN 118, HIE 144, LYS 167, GLU 183 ARG 170, ARG 216	-8.97	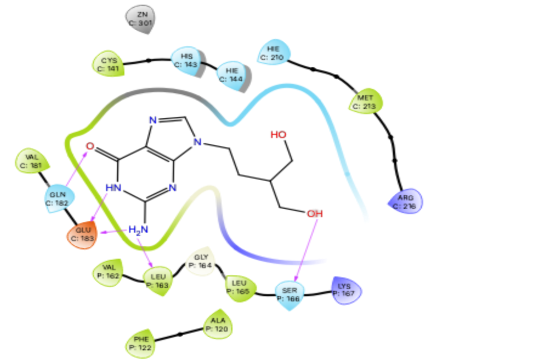
Fludarabin phosphate	anti-neoplastic	H bonds, salt bridge, Pi stacking, Pi cation, hydrophobic ASN 118, HIE 144, LYS 167, GLU 183 ARG 170, ARG 216	-9.39	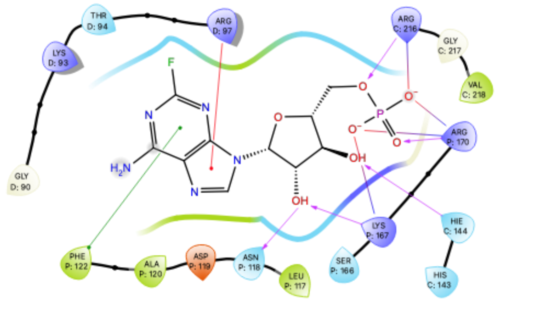
Tenofovir	Antiviral	H bonds, salt bridge, Pi stacking, Pi cation, hydrophobic ARG 97, ARG 170, HIE 210, ARG 216	-9.14	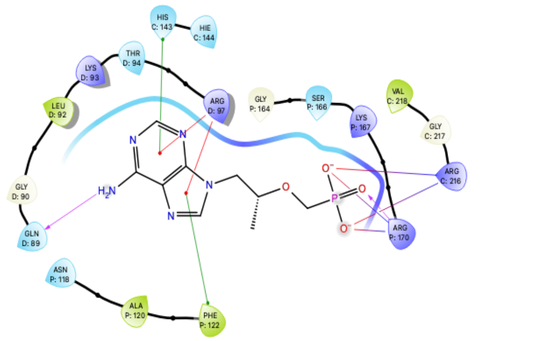
Eckol	Natural product	H bonds, Pi stacking, hydrophobic bonds. ARG 97, ASN 118, HIE 144,	-8.59	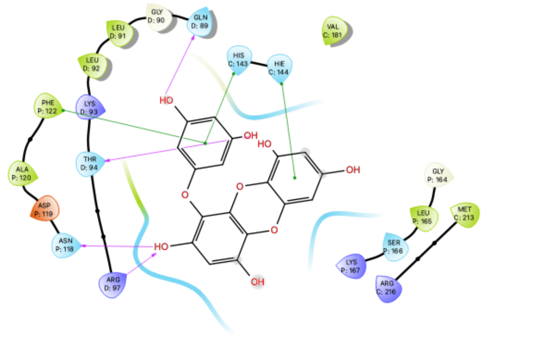

Inosine	Nucleoside /supplement	H bonds, hydrophobic SER 166, GLN 182, GLU 183, HIE 210	-8.45	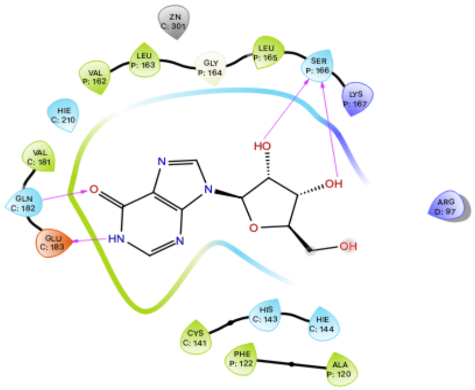
Valganciclovir	Antiviral	H bonds, hydrophobic ASN 118, HIE 144, SER 166, LEU 163, LYS 167, ARG 170, GLN 182, GLU 183, HIE 210	-8.34	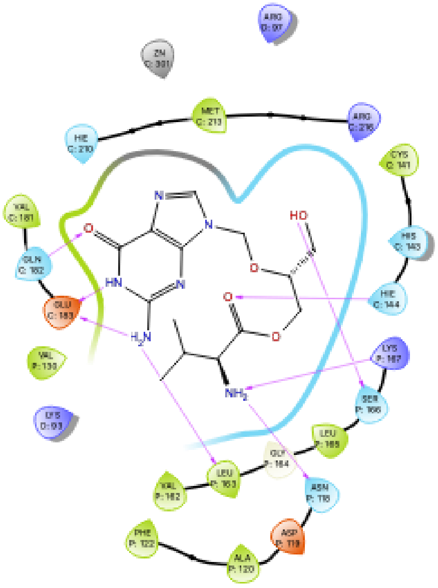
Fosfomycin	Antibiotic	H bonds, salt bridge, hydrophobic ASN 118, HIE 144, SER 166, LEU 163, LYS 167, ARG 170, GLN 182, GLU 183, HIE 210, ARG 216	-7.85	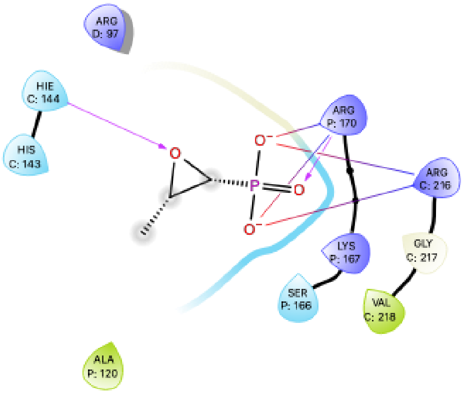
Olsalazine	Anti-inflammatory	H bonds, salt bridge, Pi stacking, hydrophobic ASN 118, HIE 144, SER 166, LEU 163, LYS 167, ARG 170,	-7.53	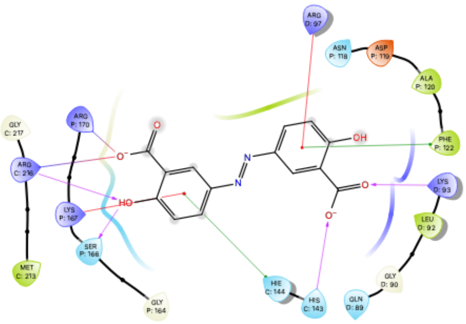

		GLN 182, GLU 183, HIE 210, ARG 216		
Phenytoin catechol	Metabolite of phenytoin antiepileptic	H bonds, Pi stacking, hydrophobic bonds and salt bridges ARG 97, HIE 144, SER 166, LYS 167	-7.19	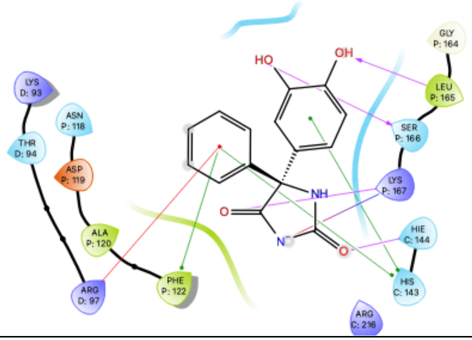
Vaborbactam	Antibiotic	H bonds, salt bridge, hydrophobic SER 166, LYS 167, ARG 170, ARG 216	-7.10	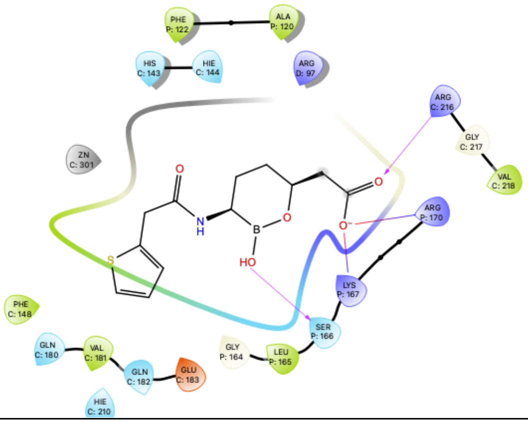

Table S3: Docking scores and 2D poses of identified hit compounds screening against the GCH1 active site.

Properties	Parameters	Olsalazine	Phenytoin catechol	Phlorofucofuroeckol	Eckol	Inosine	Valganciclovir
Physio-chemical	MW (g/mol)	302.24	284.27	602.46	372.28	268.23	390.39
	Heavy atoms	22	21	44	27	19	28
	Aromatic heavy atoms	12	12	31	18	9	12
	H-bond acceptors	8	4	14	9	7	8
	H-bond donors	4	4	9	6	4	6
Lipophilicity	Log $P_{o/w}$	2.01	1.05	3.24	1.83	-1.56	0.62
Water Solubility	Log S (ESOL)	-3.83 Soluble	-2.74 Soluble	-6.77 Poorly soluble	-4.06 Moderately soluble	-0.90 Very soluble	-2.90 Soluble
PK	GI absorption	Low-Moderate	High	Low	Moderate	Moderate	Low
	BBB permeability	nil	nil	nil	nil	nil	nil
	CYTP450 inhibitor	No	No	Yes CYP2C9	Yes CYP2C9	No	No
	Bioavailability score	0.56	0.55	0.17	0.55	0.55	0.55
Toxicity	Hepatotoxicity	+	+	nil	+	nil	nil
	Carcinogenicity	nil	nil	nil	nil	nil	nil
Status	Clinically approved	Yes	Yes			Yes	Yes
	Experimental (clinical)			Yes	Yes		

Table S4: ADMET profile for the top six hit drugs and naturaceuticals. Verified and predicted values derived from PubChem database and using SwissADME and Schrodinger's ADME/Tox prediction tool.

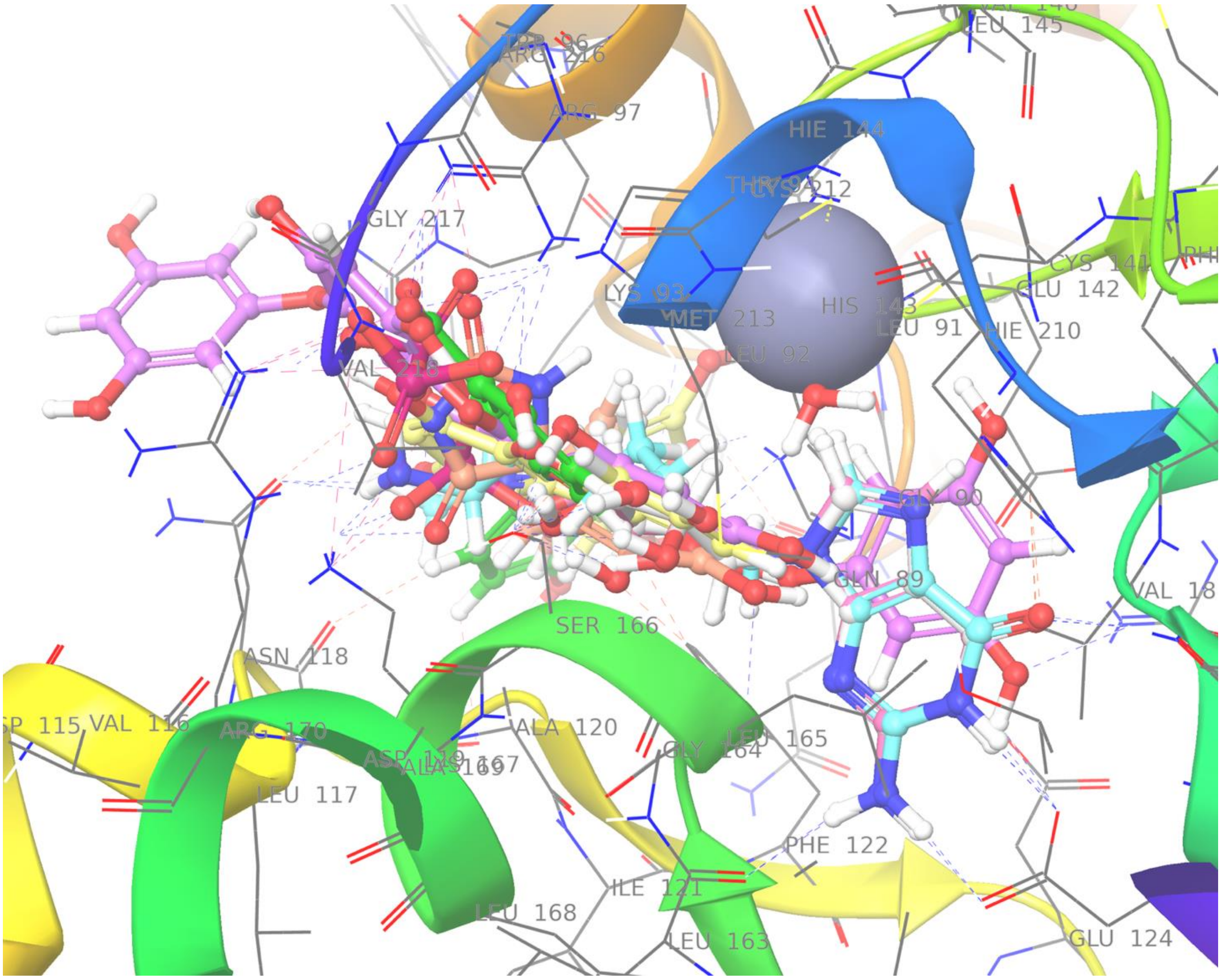

Figure S1: All fifteen hit compounds docked superimposed in the GTPCH1 binding pocket. Interactions are shown as dotted lines.

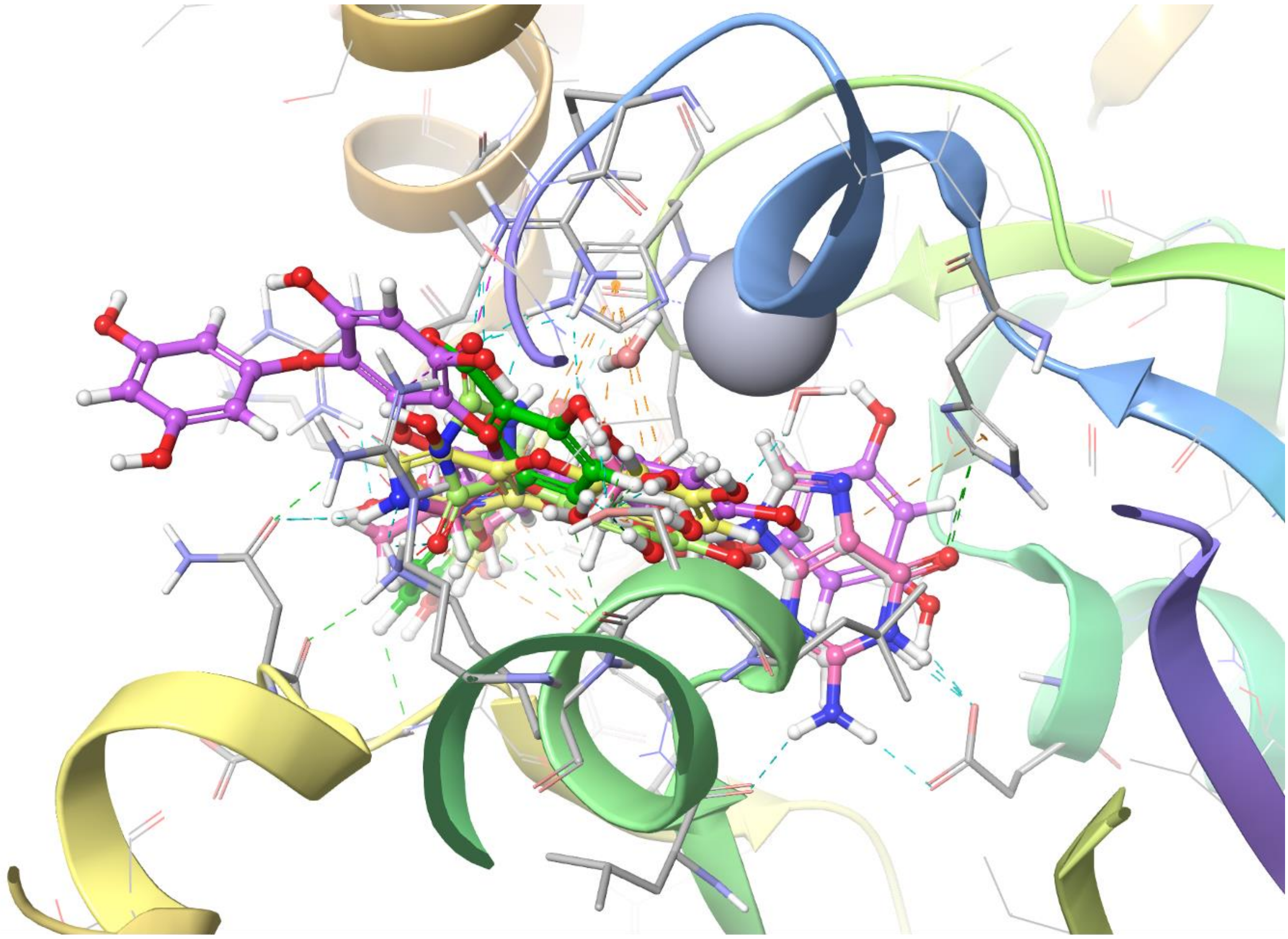

Figure S2: All six hit compounds and the control ligand docked and superimposed in the GTPCH1 binding pocket. Interactions are shown as dotted lines. Binding residues are depicted in grey bold wire, zinc ion as a grey sphere.

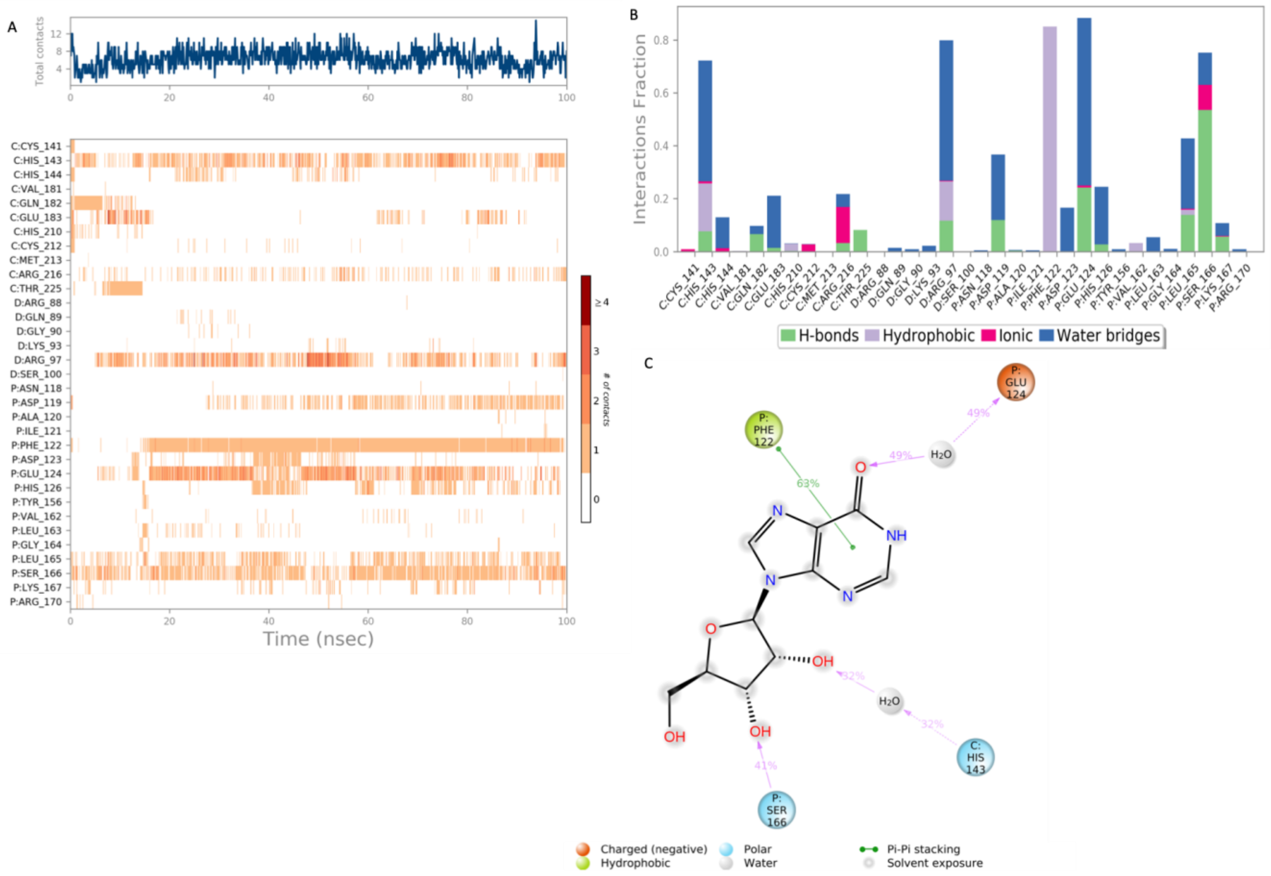

Figure S3: Interaction diagram of inosine with the GTPCH1 binding pocket. (A) Interaction of inosine with residues in each trajectory frame. The depth of color indicating the higher the interaction with contact residues; (B) The protein-ligand contacts showing the bonding interactions fraction and the nature of the interactions; (C) Graphical 2D illustration of inosine interacting with the protein residues during MD simulation. Interactions shown are occurring more than 30% during the simulation time. C: chain C of the GTPCH1 binding pocket, P: chain P of the binding pocket.

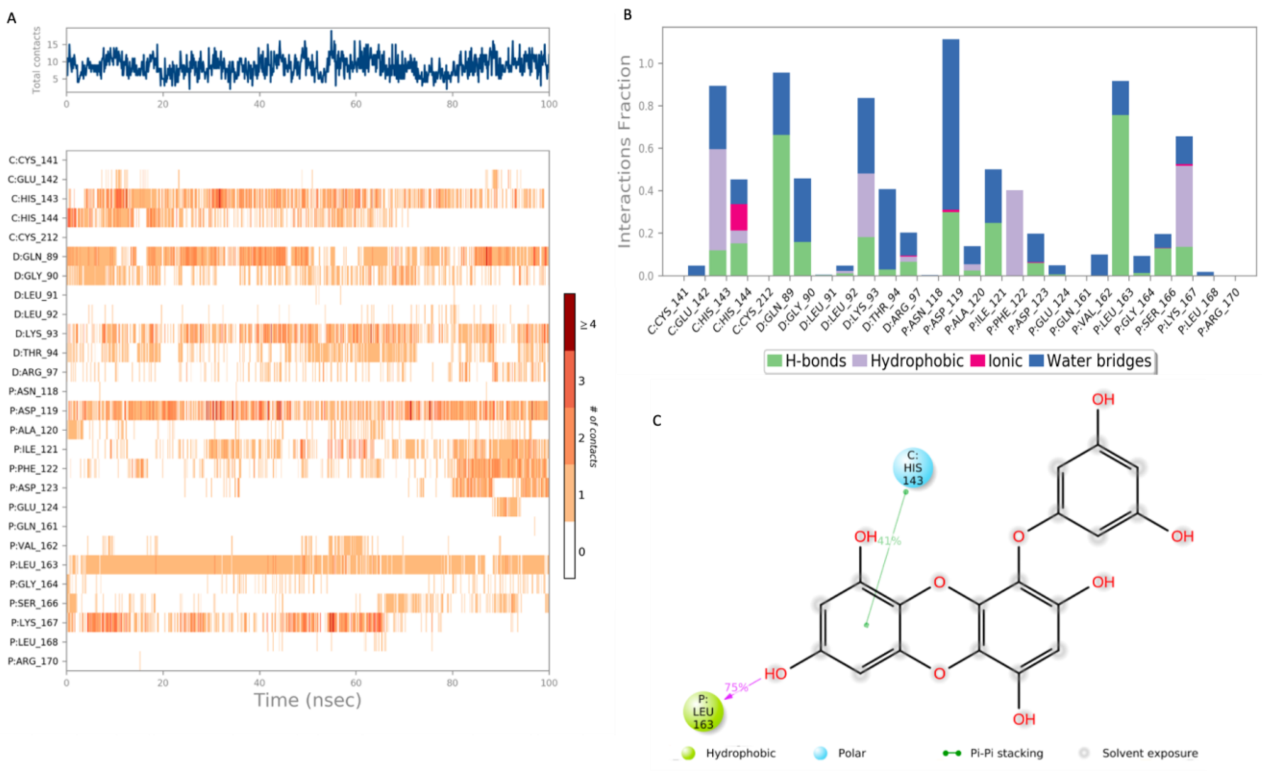

Figure S4: Interaction diagram of eckol with the GTPCH1 binding pocket. (A) Interaction of eckol with residues in each trajectory frame. The depth of color indicating the higher the interaction with contact residues; (B) The protein-ligand contacts showing the bonding interactions fraction and the nature of the interactions; (C) Graphical 2D illustration of eckol interacting with the protein residues during MD simulation. Interactions shown are occurring more than 30% during the simulation time. C: chain C of the GTPCH1 binding pocket, P: chain P of the binding pocket.

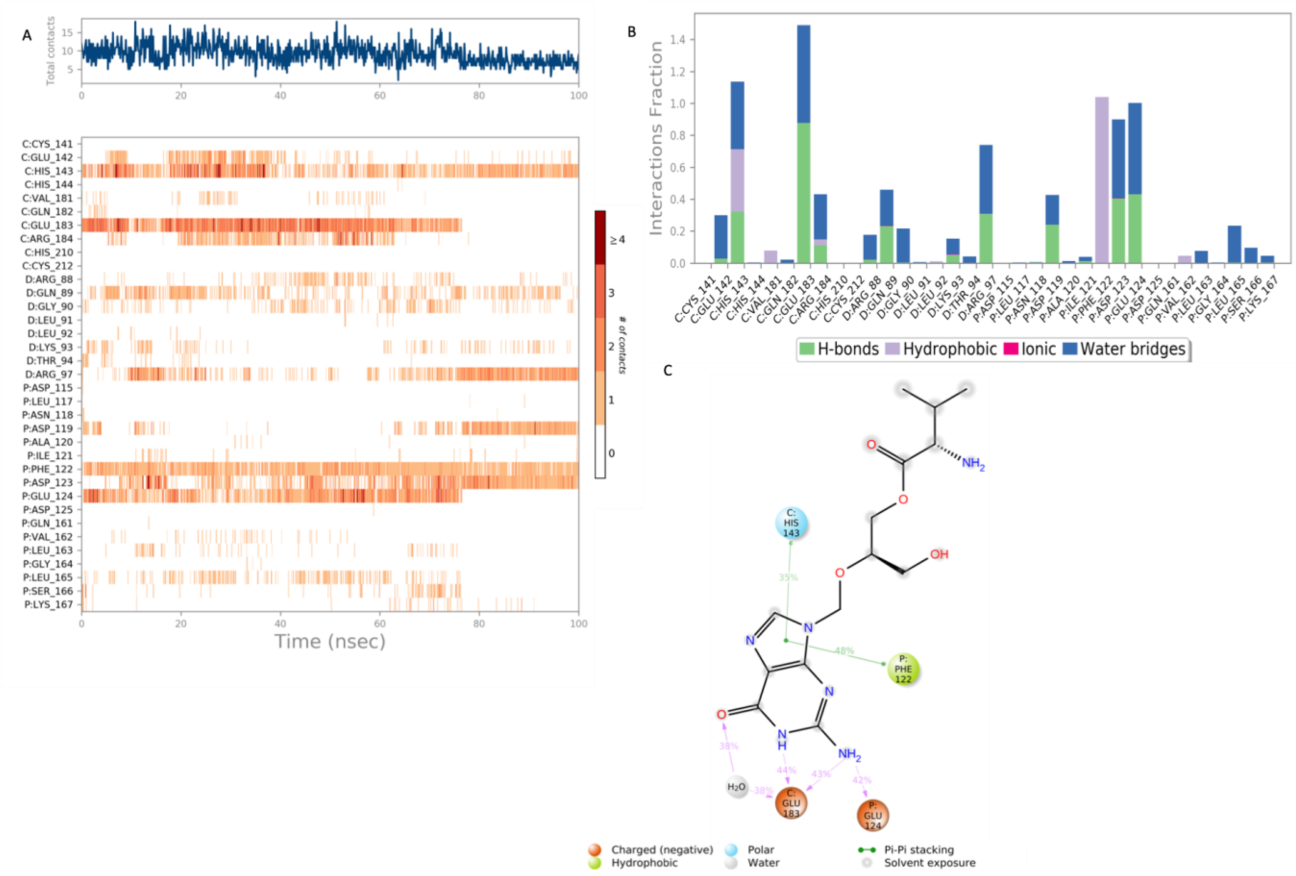

Figure S5: Interaction diagram of valganciclovir with the GTPCH1 binding pocket. (A) Interaction of valganciclovir with residues in each trajectory frame. The depth of color indicating the higher the interaction with contact residues; (B) The protein-ligand contacts showing the bonding interactions fraction and the nature of the interactions; (C) Graphical 2D illustration of valganciclovir interacting with the protein residues during MD simulation. Interactions shown are occurring more than 30% during the simulation time. C: chain C of the GTPCH1 binding pocket, P: chain P of the binding pocket.
